# Protein structure prediction powered by artificial intelligence: from biochemical foundations to practical applications

**DOI:** 10.3389/fmolb.2026.1767821

**Published:** 2026-03-09

**Authors:** Tianxiang Yin, Yunxuan Chen, Yuhang Wang, Hongyu Su, Chengxu Duan, Jingrui Liu

**Affiliations:** 1 Department of Health Technology and Informatics, The Hong Kong Polytechnic University, Hong Kong SAR, China; 2 Zonglian College, Xi’an Jiaotong University, Xi’an, Shaanxi, China; 3 College of Optoelectronic Engineering, Chongqing University, Chongqing, China; 4 West China Hospital, Sichuan University, Chengdu, Sichuan, China; 5 School of Mechanical Engineering, Xi’an Jiaotong University, Xi’an, Shaanxi, China; 6 College of Engineering and Applied Science, University of Cincinnati, Cincinnati, OH, United States

**Keywords:** AlphaFold, artificial intelligence, ESMFold, protein language models, protein structure prediction, RoseTTAFold

## Abstract

The three-dimensional structure of a protein underpins its biological function, making structure determination and prediction central challenges in structural biology. Although experimental techniques such as X-ray crystallography, nuclear magnetic resonance (NMR), and cryo-electron microscopy (cryo-EM) can yield high-resolution structures, they are limited by low throughput, high cost, and demanding sample preparation. Likewise, traditional computational methods often perform poorly in the absence of homologous templates or under complex folding dynamics. Recent advances in deep learning and large-scale protein language models have transformed protein structure prediction. Models such as AlphaFold3 and RoseTTAFold achieve near-experimental accuracy by integrating evolutionary information, geometric constraints, and end-to-end neural architectures, while single-sequence approaches such as ESMFold offer substantial gains in speed and scalability. This review summarizes the biochemical foundations of protein folding, recent AI-driven methodological advances, and representative applications in drug discovery, enzyme engineering, and disease research, and discusses current challenges and future directions.

## 1 Introduction

**FIGURE 1:**
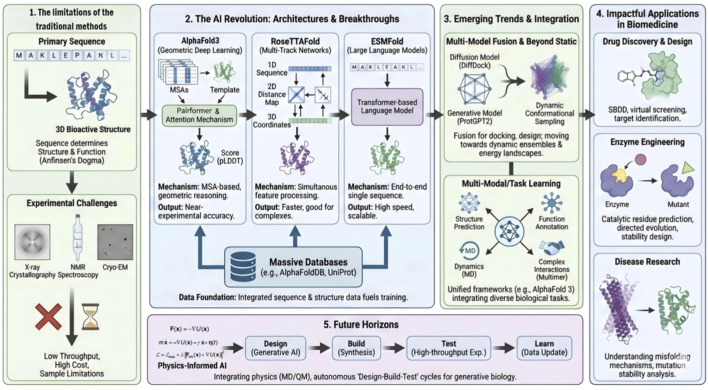
Overview of AI-based protein structure prediction frameworks and emerging trends.

Proteins are fundamental structural and functional units of living systems, and their three-dimensional conformations largely determine biological activity and specificity. Protein misfolding or structural distortion is closely associated with numerous diseases, including Alzheimer’s disease and cystic fibrosis (Khan and Khan, 2022; Patton-Parfyonov et al., 2025). Over past decades, advances in structural biology—most notably X-ray crystallography (Russell et al., 2004), nuclear magnetic resonance (NMR) spectroscopy (Russell et al., 2004), and cryo-electron microscopy (cryo-EM) (Eisenstein, 2025)—have enabled detailed investigations of protein structures and have driven progress in molecular biology, medicinal chemistry, and enzymology. X-ray crystallography can achieve atomic-level resolution but requires high-quality crystals, which remain difficult to obtain for many proteins, particularly membrane proteins, large macromolecular complexes, or intrinsically flexible systems. NMR spectroscopy is well suited for determining protein structures in solution, yet its applicability is generally limited to proteins smaller than approximately 30 kDa (∼270 amino acids) due to spectral overlap and signal attenuation. Cryo-EM has emerged as a powerful technique for studying large macromolecular assemblies, benefiting from recent improvements in detector technology and image processing; however, it remains costly and faces challenges related to sample preparation, image complexity, and resolution for flexible regions. Collectively, these experimental approaches are characterized by low throughput, high cost, and restricted applicability. In contrast, the growth of protein sequence databases has far outpaced that of experimentally resolved structures, creating an urgent need for high-throughput, cost-effective structure prediction methods. Artificial intelligence, particularly deep learning, is rapidly transforming the field of protein structure prediction. AlphaFold2 achieved near-experimental accuracy for approximately two-thirds of the targets in the CASP14 competition (Lupas et al., 2021), and its developers subsequently reported confident structural coverage (pLDDT >70) for approximately 98.5% of the human proteome, though not all regions within these models exhibit high confidence (Tunyasuvunakool et al., 2021). Recent reviews further highlight deep learning-based protein structure prediction and design as a major research frontier (Hou et al., 2025; Liu et al., 2025). The overall organization and key topics discussed in this article are summarized in Figure 1.

## 2 Traditional methods for determining protein structures

The primary structure of a protein consists of a specific amino acid sequence, which folds into secondary, tertiary, and quaternary structures through a range of intra- and intermolecular physicochemical interactions. According to Anfinsen’s thermodynamic hypothesis (Gambardella et al., 2022), the native folded state of a protein is determined primarily by its amino acid sequence, corresponding to the global minimum of free energy under physiological conditions.

Common secondary structure elements include α-helices and β-sheets, while tertiary structure formation arises from the collective effects of hydrophobic interactions, hydrogen bonding, van der Waals forces, and electrostatic interactions. Hydrophobic residues tend to be buried within the protein core to minimize solvent exposure, whereas hydrophilic residues are generally exposed on the surface to reduce free energy (Dill, 1990). Hydrogen bonds stabilize secondary structures by constraining the protein backbone, van der Waals interactions provide fine-scale packing stability, and electrostatic interactions—including ionic bonds and dipole interactions—are particularly important in active sites and surface recognition regions. For proteins composed of multiple subunits, quaternary structures arise from subunit assembly, further increasing structural complexity and functional diversity.

Traditional structure determination methods rely on experimental techniques such as X-ray crystallography, which reconstructs electron density maps from diffraction data but requires crystalline samples and is often time-consuming with high failure rates. NMR spectroscopy is suitable for small proteins in solution but is limited by molecular size and spectral complexity. Cryo-EM involves imaging vitrified samples at cryogenic temperatures and has rapidly expanded its scope, though challenges remain in sample heterogeneity, data processing, and resolving flexible regions.

Classical computational approaches to structure prediction include homology modeling, fold recognition (threading), and *ab initio* modeling. Template-based methods perform well when closely related homologous structures are available, but their accuracy deteriorates for novel or orphan proteins lacking structural analogs. Ab initio approaches, which include physics-based simulations (e.g., molecular dynamics) and fragment-assembly methods (e.g., Rosetta), are theoretically general in the sense that they do not rely on homologous templates and can, in principle, predict structures for any protein sequence. However, they are computationally expensive and often exhibit limited accuracy in practice, especially for larger proteins, due to challenges in conformational sampling, force field approximations, and the vastness of the folding landscape. Overall, traditional experimental methods are low throughput, while conventional computational techniques face scalability and precision limitations, underscoring the transformative potential of AI-based approaches (Zheng et al., 2025; Lee et al., 2023; Dauparas et al., 2022).

## 3 Breakthroughs in AI-based protein structure prediction

In recent years, several AI-based architectures have emerged for protein structure prediction. These include: integrating physical and biological knowledge of protein structures with multiple sequence alignments (MSA) to design deep learning algorithms that capture geometric information (AlphaFold Series) (Jumper et al., 2021); employing three-track or multi-scale networks to learn sequence–distance–coordinate representations (RoseTTAFold) (Abramson et al., 2024); and using large-scale protein language models for end-to-end single-sequence prediction (ESMFold/ESM-2) (Lin et al., 2023). The predictive capability of AlphaFold3 is rooted in its next-generation architecture, which includes an improved version of the Evoformer module. This deep learning architecture played a crucial role in the success of its predecessor, AlphaFold2, and has been further optimized to enhance performance. The model employs a diffusion network process, starting from an atomic cloud and gradually converging to the most accurate molecular structure. This approach enables AlphaFold3 to generate the joint three-dimensional (3D) structure of the input molecules, revealing their overall compatibility (Abramson et al., 2024). RoseTTAFold, on the other hand, employs a three-track network to transform and integrate one-dimensional sequences, two-dimensional distance matrices, and three-dimensional coordinate layers, enabling faster and more generalizable predictions (Patton-Parfyonov et al., 2025). In the language model-based approach, some methods combine protein language model representations with natural polypeptide geometric descriptions to efficiently explore sequence–structure relationships, as exemplified by RGN2 (Lin et al., 2023).

Regarding predictive performance, AlphaFold3 achieves atomic-level accuracy in single-structure prediction, and exhibits faster inference speed and performs more reliably on protein–protein interactions, multi-domain proteins, mutated or post-translationally modified proteins, as well as orphan proteins lacking homologous sequences than AlphaFold2 (Khan and Khan, 2022; Eisenstein, 2025; Abramson et al., 2024). One of the most significant advancements of AlphaFold three is its expanded predictive capability. The model is now capable of accurately predicting protein-molecule complexes that include various biological molecules such as DNA and RNA. This expansion is crucial for applications in genomics research and drug discovery, providing researchers with a powerful tool for more rapid and accurate predictions (Abramson et al., 2024). Compared with AlphaFold3, RoseTTAFold demonstrates superior performance in predicting mutated protein structures and offers faster prediction, albeit with slightly lower accuracy (Yang et al., 2023). Language model-based approaches, led by RGN, achieve orders-of-magnitude improvements in prediction speed and perform better on orphan proteins lacking homologous sequences; however, these models suffer from lower interpretability and reliability, and the correctness of their predictions remains uncertain (Chowdhury et al., 2022). Overall, these models have advanced the performance of template-free predictions, marking a breakthrough from known-to-unknown protein structure inference. Furthermore, they have promoted transparency in structural information through databases and open-source platforms (Service, 2021). These models are summarized in Table 1. It is important to note that protein structure prediction, protein–ligand docking, protein design, database construction, and evaluation tools represent distinct methodological tasks rather than a single continuous pipeline. While AI-based structure prediction can facilitate downstream analyses, each step involves different assumptions, sources of uncertainty, and validation requirements. Treating these approaches as a unified workflow may overstate the current capabilities of the field.

**TABLE 1:** Selected AI-based methods and resources related to protein structure prediction and downstream analysis.

Model/Method	Source	Core architecture/Innovation	Advantages	Applications/Directions
AlphaFold Series	Jumper et al. (2021), Yang et al. (2023), Stahl et al. (2023), Hekkelman et al. (2023), Wayment-Steele et al. (2024), Abramson et al. (2024)	AF2: Combines MSA and structural modules; AlphaLink: Integrates crosslinking MS data; AlphaFill: Transplants small molecule ligands; AF-Cluster: Predicts multiple conformations via MSA clustering; AlphaFold 3: Unified diffusion-based model	Atomic-level accuracy, integration of experimental data, ligand transplantation, multi-conformation prediction, and multi-component complex modeling	Large-scale structural prediction, experimental integration, multi-component complex modeling, drug discovery platform
RoseTTAFold	Abramson et al. (2024)	Three-track network integrating 1D sequence, 2D distance map, and 3D coordinate information	High speed, suitable for protein complexes and mutation prediction, strong generalization	Protein–protein complex prediction, X-ray/cryo-EM-assisted modeling
Protein Language Model Series	Lin et al. (2023), Chowdhury et al. (2022), Wang et al. (2024), Nijkamp et al. (2023), Ferruz et al. (2022), Callaway (2022)	ESMFold: Large-scale language model for end-to-end prediction; RGN2: Language model + geometric representation; ProT-Diff: Language model + diffusion generation; ProGen2/ProtGPT2: Large-scale generative language models	Extremely fast prediction, suitable for orphan proteins, no MSA required, supports sequence generation and design	Metagenomic structural survey, protein design and engineering, antimicrobial peptide discovery
AlphaFold (Initial)	Senior et al. (2020)	Distance-based deep learning method constructing residue–residue distance maps	Strong performance in CASP13, significant improvement in template-free prediction	Milestone in early AI-based structure prediction
Evaluation and Tool Platforms	Buttenschoen et al. (2024), Michel et al. (2024), Lau et al. (2024), Van Kempen et al. (2024)	PoseBusters: Physical plausibility assessment; Foldseek: Fast structural alignment; TED: Domain parsing and classification	Standardized evaluation framework, high-speed search tools, structural domain resource integration	Method standardization, large-scale data analysis, domain evolution research
Databases and Resources	Pándy-Szekeres et al. (2024), Tunyasuvunakool (2022)	GproteinDb: GPCR–G protein complex database; CASP14 impact review	Provides structural templates and coupling data, promotes routine use of structure-guided experiments	GPCR research, experimental–computational integration, structural biology toolset development
Protein–Ligand Docking Methods	Cao et al. (2025)	SurfDock: Surface-informed diffusion generative model	High docking success rate and physical plausibility, adaptable to unseen proteins and predicted apo structures	Drug discovery, virtual screening, protein–ligand interaction studies

Unlike the early single-method prediction algorithms, modern approaches not only integrate homology-based sequence alignment methods with *ab initio* modeling guided by physical laws but also develop deep learning models on this basis (Yang et al., 2023; Senior et al., 2020). These models can automatically learn the sequence–structure mapping while capturing long-range residue dependencies and co-evolutionary features through attention mechanisms. For example, AlphaFold three employs a unique iterative refinement technique called “recycling”, which significantly enhances its accuracy. This process involves repeatedly applying the final loss to the output and then recursively feeding it back into the network (Abramson et al., 2024). This method enables continuous refinement and development of highly accurate protein structures with precise atomic details. Similarly, the multi-track network in RoseTTAFold overcomes the limitations of conventional single-channel neural networks in modeling spatial geometry, with the three-dimensional structure representation layer improving residue–residue angle prediction accuracy (Abramson et al., 2024).

In recent years, multi-model fusion and generative architectures have further expanded structural prediction. Diffusion-based models such as DiffDock and ProtDiffusion explore conformational space via diffusion sampling, providing new approaches for protein–ligand complex prediction and protein design (Buttenschoen et al., 2024; Wang et al., 2024). Beyond diffusion models, alternative generative frameworks are also emerging. For instance, the Boltz series (e.g., Boltz-1, Boltz-2 and BoltzGen) from MIT employs a full-atom co-folding approach trained on structural data. These models offer the distinct advantage of co-folding a protein with a user-specified ligand while simultaneously predicting binding affinities with high accuracy. Moreover, large language models, including Meta AI ESMFold, ProGen2, and Salesforce ProtGPT2, demonstrate capabilities to infer structure from sequence and predict function from structure, blurring the boundary between structure prediction and protein design (Nijkamp et al., 2023; Ferruz et al., 2022). Multi-model fusion manifests not only in multiple models performing the same function but also in the refinement of individual models to achieve multifunctional integration. Enhancing existing models has become a trend in the development of multifunctional frameworks. For instance, following the release of AlphaFold2, AlphaLink integrates experimental distance constraints to improve prediction accuracy (Stahl et al., 2023); AlphaFill enriches AlphaFold models with ligands and cofactors, supplementing small-molecule coordinates (Hekkelman et al., 2023); and AF-Cluster performs multiple-conformation prediction by clustering MSAs based on sequence similarity (Wayment-Steele et al., 2024).

A second trend is multi-modal, multi-task learning. Structural prediction is increasingly integrated with function annotation, dynamics simulation, and position-based recognition within unified network frameworks. Notable examples include AlphaFold 3, UniFold, and DeepMind AlphaFold-Multimer, which enable breakthroughs in complex structure prediction and binding energy calculation (Abramson et al., 2024; Pándy-Szekeres et al., 2024). Such models provide a reliable computational foundation for predicting protein–protein and protein–nucleic acid interactions (Stahl et al., 2023). Significant progress has been made with the release of AlphaFold 3, which employs a unified diffusion-based architecture to directly model the joint structure of biomolecular complexes. It shows improved accuracy in predicting protein-protein and protein-nucleic acid interactions, and shows enhanced capability in handling multi-domain proteins. However, challenges remain, particularly in capturing the full conformational dynamics of interfaces and in the prediction of structures involving small molecules or post-translational modifications without explicit templates. Overall, AI-based protein structure prediction has evolved from “monomeric folding” toward “system-level modeling,” integrating generative models, molecular dynamics, and language models. This development suggests that structure prediction will not be limited to reproducing static conformations but will advance toward dynamic conformational sampling, energy landscape exploration, and function-guided design (Tunyasuvunakool, 2022).

A third trend is the critical role of databases and the high demand for information integration and exchange. Unlike early physics-based structural prediction algorithms, modern AI architectures—such as AlphaFold3, RoseTTAFold, and ESMFold—require extensive data extraction and integration prior to deep learning and model training. Large-scale amino acid sequence and structural datasets are a key factor behind AlphaFold2 success, enabling the exploration of sequence–structure dependencies and information exchange within MSAs to capture co-evolutionary signals (Yang et al., 2023). RoseTTAFold incorporates cropped MSAs, allowing information flow across the three tracks to enable systematic structural reasoning (Abramson et al., 2024). ESMFold relies on extensive textual data to train protein language models, achieving sequence–structure interaction via end-to-end single-sequence predictors. In summary, AI models for protein structure prediction depend on robust database support and comprehensive information integration. Current platforms, such as the Encyclopedia of Protein Domains and Foldseek, facilitate management and analysis of predicted protein models (Michel et al., 2024; Callaway, 2022; Lau et al., 2024; Van Kempen et al., 2024), not only enabling resource sharing across biology, medicine, and other disciplines but also promoting database expansion, model updates, and iterative improvements. Future development of protein structure prediction models will impose even higher requirements on databases and deep learning frameworks for information integration (Cao et al., 2025).

## 4 Applications of artificial intelligence in biochemistry and medicine

Breakthroughs in artificial intelligence (AI)-driven protein structure prediction have opened up broad prospects for biological and medical research. Accurate structural information enables in-depth analysis of protein functions and interaction mechanisms, while also guiding and accelerating experimental workflows. As a result, drug discovery and enzymatic mechanism studies can be conducted at lower cost, higher speed, and improved precision, facilitating the rational design of therapeutics and the exploration of disease mechanisms (Yang et al., 2023).

Despite the rapid adoption of AI-based protein structure prediction tools, it is essential to recognize their limitations before applying predicted models to downstream tasks. Current methods typically generate a single static conformation and do not capture the conformational ensembles that proteins often populate in solution. Moreover, prediction confidence metrics (e.g., pLDDT) primarily reflect internal model consistency and should not be interpreted as guarantees of functional accuracy, interaction fidelity, or binding-site correctness. As a result, AI-predicted structures should not be blindly trusted in applications such as drug discovery or enzyme engineering. Instead, they should be treated as hypothesis-generating models that require expert interpretation, careful refinement, and integration with experimental data.

In drug discovery, AI-based protein structure prediction substantially improves the accuracy of target identification and virtual screening. For example, AlphaFold2 is capable of generating near-experimental-level structural predictions within a short time frame. By deeply embedding evolutionary information and amino acid sequences, its Evoformer module processes both intra-sequence and inter-sequence relationships, while the structure module iteratively refines a three-dimensional protein model using the features extracted by the Evoformer. The final output is a high-quality computational structural model that often captures the overall fold and domain organization of the target protein. However, such models should be regarded as predictions rather than experimentally determined atomic-resolution structures, and they typically require further refinement and experimental validation before being used in structure-based drug design (Skolnick et al., 2021). In particular, inaccuracies are frequently observed in loop regions, side-chain conformations, flexible domains, and binding-site geometry, and predicted models generally lack ligands, cofactors, structured water molecules, and information on alternative conformational states, all of which are critical for reliable SBDD. With continued advances, protein structure prediction is evolving from a “computational service” into a global scientific “infrastructure.” A representative example is the AlphaFold Protein Structure Database (AlphaFoldDB), jointly developed by DeepMind and the European Molecular Biology Laboratory - European Bioinformatics Institute (EMBL-EBI). This open and freely accessible resource contains precomputed structures for a vast number of proteins, analogous to public genome sequence repositories. AlphaFoldDB (Varadi et al., 2022) currently includes over 214 million predicted structures, covering a wide range of organisms and metagenomic samples. By providing atomic coordinates, predicted Local Distance Difference Test (pLDDT) scores, and predicted alignment error maps, the database has been widely adopted in medicinal chemistry, virology, and structural immunology. While many regions exhibit high confidence (pLDDT >70), substantial portions of some predictions may have low confidence scores, reflecting inherent prediction uncertainty. Importantly, commonly reported confidence scores such as pLDDT assess local structural consistency rather than functional correctness, and high-confidence predictions may still be unreliable for modeling protein–ligand interactions, interface geometry, or dynamic regions. It is worth noting that some studies directly use structures predicted by AlphaFold3 for molecular docking without considering conformational flexibility, binding pocket deformation, or ligand-induced conformational changes, resulting in lower hit rates in virtual screening. Therefore, in high-risk applications (such as antibody design or targeted therapy development), it is recommended to incorporate experimental constraints, conformational sampling, or multiple model integration to enhance the reliability of predictions. In antibody engineering and protein-protein interaction prediction, models such as RoseTTAFold and AlphaFold-Multimer enable more accurate structural modeling of protein complexes, significantly enhancing predictive capabilities in biopharmaceutical research (Baek et al., 2024; Tang et al., 2025).

AI has also played a transformative role in enzyme engineering and directed evolution. By integrating natural and artificially designed enzymes, researchers have employed ESMFold, developed by Meta AI, to rapidly predict high-accuracy three-dimensional enzyme structures. Geometric graph neural networks (GNNs) and attention-based models are then applied to focus on a limited number of key nodes—specific amino acid residues—predicted to be catalytic residues, thereby guiding experimental selection and mutation screening (Pancino et al., 2024; Lin et al., 2023). In addition, molecular dynamics (MD) simulations are used to investigate the dynamic behavior of enzyme active sites, including the stability of substrate-binding pockets and interactions among critical residues, which can further inform the targeted screening of high-activity mutants (Ito et al., 2025). AI methods have also been applied to mutation prediction to assist researchers in selecting more stable or more soluble enzymes. ProteinMPNN, a novel protein sequence design method based on message passing neural networks, outperforms previous mainstream approaches in terms of speed, accuracy, and success rate through innovations in network architecture and training strategies. ProteinMPNN can directly generate highly foldable, stable, and soluble sequences and is currently regarded as one of the most powerful tools for *de novo* design of stable enzyme sequences (Sumida et al., 2024). Nevertheless, inaccuracies in predicted active-site geometry and the absence of conformational dynamics remain major challenges, and experimental validation remains indispensable for confirming catalytic mechanisms and functional effects of designed mutations.

In disease research, structure-prediction AI facilitates the understanding of pathogenic protein misfolding and mutations. For instance, the structural features of amyloid-β (Aβ) aggregates associated with Alzheimer disease have been investigated by combining AlphaFold2 predictions with molecular dynamics simulations to explore different β-sheet arrangements induced by mutations. Similarly, AI-predicted structures enable rapid investigation of mutation-induced stability changes in tumor-related proteins such as p53, KRAS, and BRCA1, thereby contributing to mechanistic insights into disease progression and supporting the development of targeted and therapeutic drugs (Dauparas et al., 2022).

Beyond individual applications, the openness and automation of AI-based structure prediction are reshaping the research ecosystem. Major pharmaceutical companies, including Pfizer, Novartis, and AstraZeneca, have begun integrating AlphaFold and RoseTTAFold models into their internal drug discovery pipelines to automate structure prediction, molecular docking, and candidate compound screening (Callaway, 2025; Varadi and Velankar, 2022). By combining AI-derived predictions with experimental validation techniques—such as cryo-electron microscopy, *in vivo* cross-linking mass spectrometry, and NMR-based restraints—an integrated *in vitro* experimental design paradigm is emerging (Khan et al., 2024; Bley et al., 2022). Collectively, these developments demonstrate that AI has become a foundational tool in modern structural biochemistry and precision medicine.

## 5 Conclusion

AI-driven protein structure prediction represents a turning point in structural biology. The transition from physics-based energy modeling to deep learning-based sequence-structure inference has yielded dramatic improvements in both accuracy and speed. Models such as AlphaFold, RoseTTAFold, and ESMFold demonstrate the capacity of deep learning to generalize molecular reasoning across complex biological systems.

Despite these advances, significant challenges remain. Current models typically output a single static conformation, whereas proteins *in vivo* populate dynamic conformational ensembles. Protein-ligand and protein-nucleic acid interaction prediction remains in an early stage, and model interpretability and reliability are not yet sufficient to replace experimental validation. Additionally, dataset bias and imbalance—particularly underrepresentation of membrane proteins and intrinsically disordered regions—continue to limit generalization.

Future research directions include (Zheng et al., 2025; Vignesh et al., 2024; Mao et al., 2025): (i) tighter integration with physical modeling through molecular dynamics and quantum chemical methods to unify structure, energy, and dynamics; (ii) development of multi-state prediction frameworks incorporating sequence, structure, function, and experimental constraints to enhance interpretability; and (iii) establishment of automated design-validation-optimization pipelines enabling generative protein engineering.

With the emergence of models such as AlphaFold3 and rapid advances in generative protein models, AI-driven structure prediction is poised to evolve from static reconstruction toward dynamic understanding, providing foundational support for precision medicine, synthetic biology, and intelligent drug discovery.
